# Canakinumab (ACZ885, a fully human IgG1 anti-IL-1β mAb) induces sustained remission in pediatric patients with cryopyrin-associated periodic syndrome (CAPS)

**DOI:** 10.1186/ar3266

**Published:** 2011-02-28

**Authors:** Jasmin B Kuemmerle-Deschner, Eduardo Ramos, Norbert Blank, Joachim Roesler, Sandra D Felix, Thomas Jung, Kirstin Stricker, Abhijit Chakraborty, Stacey Tannenbaum, Andrew M Wright, Christiane Rordorf

**Affiliations:** 1 Division of Pediatric Rheumatology, Department of Pediatrics, University Children's Hospital Tuebingen, Hoppe-Seyler-Strasse 1, 72076 Tübingen, Tuebingen, Germany; 2 Department of Pediatrics, Hospital Central de Asturias, Julian Claveria s/n, 33006 Oviedo, Spain; 3 Medizinische Klinik 5, Universitätsklinikum Heidelberg, Im Neuenheimer Feld 410, D-69120, Heidelberg, Germany; 4 Department of Pediatrics, Universitäts-Klinikum Carl-Gustav-Carus, Fetscherstr. 74, 01307 Kinderklinik, Dresden, Germany; 5 Translational Medicine, Novartis Institutes for BioMedical Research, Basel CH-4002, Switzerland; 6 Development, Novartis Pharma, Basel CH-4002, Switzerland; 7 Drug Metabolism and Pharmacokinetics, Novartis Institutes for BioMedical Research, East Hanover, NJ 07936-1080, USA; 8 Modeling and Simulation, Novartis Pharmaceuticals Corporation, East Hanover, NJ 07936-1080, USA; 9 Clinical Information Sciences, Novartis Pharma, Basel CH-4002, Switzerland

## Abstract

**Introduction:**

Cryopyrin-associated periodic syndrome (CAPS) represents a spectrum of three auto-inflammatory syndromes, familial cold auto-inflammatory syndrome (FCAS), Muckle-Wells syndrome (MWS), and neonatal-onset multisystem inflammatory disease/chronic infantile neurological cutaneous and articular syndrome (NOMID/CINCA) with etiology linked to mutations in the *NLRP3 *gene resulting in elevated interleukin-1β (IL-1β) release. CAPS is a rare hereditary auto-inflammatory disease, which may start early in childhood and requires a life-long treatment. Canakinumab, a fully human anti-IL-1β antibody, produces sustained selective inhibition of IL-1β. This study was conducted to assess the efficacy, safety, and pharmacokinetics of canakinumab in the treatment of pediatric CAPS patients.

**Methods:**

Seven pediatric patients (five children and two adolescents) with CAPS were enrolled in a phase II, open-label study of canakinumab in patients with CAPS. Canakinumab was administered at a dose of 2 mg/kg subcutaneously (s.c.) (for patients with body weight ≤ 40 kg) or 150 mg s.c. (for patients with body weight > 40 kg) with re-dosing upon each relapse. The primary efficacy variable was time to relapse following achievement of a complete response (defined as a global assessment of no or minimal disease activity and no or minimal rash and values for serum C-reactive protein (CRP) and/or serum amyloid A (SAA) within the normal range, < 10 mg/L).

**Results:**

All patients achieved a complete response within seven days after the first dose of canakinumab and responses were reinduced on retreatment following relapse. Improvements in symptoms were evident within 24 hours after the first dose, according to physician assessments. The estimated median time to relapse was 49 days (95% CI 29 to 68) in children who received a dose of 2 mg/kg. Canakinumab was well tolerated. One serious adverse event, vertigo, was reported, but resolved during treatment.

**Conclusions:**

Canakinumab, 2 mg/kg or 150 mg s.c., induced rapid and sustained clinical and biochemical responses in pediatric patients with CAPS.

**Trial registration number:**

ClinicalTrials.gov: NCT00487708

## Introduction

Cryopyrin-associated periodic syndrome (CAPS) comprises a spectrum of rare inherited chronic auto-inflammatory disorders including familial cold auto-inflammatory syndrome (FCAS), Muckle-Wells syndrome (MWS), neonatal onset multisystem inflammatory disease (NOMID), also known as chronic infantile neurological, cutaneous, and articular syndrome (CINCA). Common characteristics of these disorders include high-grade fever, urticarial rash, ocular manifestations such as conjunctivitis, sensorineural hearing loss and arthritis [1-5].

Onset of symptoms generally occurs early in life, especially in patients with the two more severe phenotypes, MWS, and NOMID, and these disorders are associated with developmental abnormalities and progressive worsening of clinical manifestations such as sensorineural hearing loss and sight impairment [3-5]. In addition, the high levels of the acute phase protein, serum amyloid A protein (SAA) results in AA amyloidosis in approximately a quarter of patients with MWS, leading to renal impairment. Thus initiation of treatment in childhood is important for most patients and may reduce long-term sequelae.

All three phenotypes are associated with mutations in the *NLRP3 *gene encoding cryopyrin, also known as NALP3/CIAS1 [1,6,7]. Cryopyrin is involved in the activation of interleukin (IL)-1β [8]. Mutations in *NLRP3 *are associated with over-activation of caspase-1, the enzyme which catalyses the cleavage of the precursor of IL-1β, pro-IL-1β, to generate active IL-1β in excess [9]. This suggested that IL-1β blockade might provide effective treatment for this rare disorder. Indeed studies with anakinra, a non-glycosylated form of the endogenous antagonist of the IL-1 receptor, IL-1Ra, and rilonacept, which binds to IL-1β with high affinity and thus blocks the binding of IL-1β to its receptor, have demonstrated promising therapeutic activity in patients with CAPS [10-12]. However, anakinra requires daily administration which can be difficult, especially for pediatric patients, and injections are frequently painful and can lead to injection site reactions and rash, while rilonacept is administered once weekly and is also frequently associated with injection site reactions. Both substances are not approved for the treatment of CAPS in children. There is, therefore, a need for improved anti-IL-1β therapies for the management of CAPS and other auto-inflammatory conditions driven by overproduction of IL-1β.

Canakinumab is a fully human IgG1 anti-IL-1β monoclonal antibody that binds to human IL-1β with high specificity and neutralizes the bioactivity of this cytokine [13]. It has a half-life of 21 to 28 days in adults [14] and produces rapid and sustained clinical remissions in patients with CAPS when dosed every eight weeks [15]. This paper reports efficacy, safety, and tolerability analysis of data of the seven pediatric patients (children or adolescents) out of 34 patients who were enrolled in a phase II, open-label study.

## Materials and methods

### Study design and intervention

This study involved patients (aged 4 to 75 years, body weight ≥12 and < 100 kg) with documented *NLRP3 *mutations and a clinical picture of CAPS requiring medical intervention. Patients with a very severe phenotype receiving steroid therapy could be included if they had received a stable dose for at least one week prior to the screening visit. Patients on anakinra after a washout period of 15 days post screening were allowed. Any anti-IL-1 therapy had to be discontinued before entering the study. Female patients of child bearing age were to use an effective method of contraception during the study and for at least three months after the last dose.

Patients received canakinumab at a dose of 2 mg/kg (body weight < 40 kg) or 150 mg s.c. (body weight ≥40 kg). Patients not achieving a complete response within seven days post initial s.c. treatment received a re-dose of canakinumab (5 or 10 mg/kg i.v.) as a rescue medication. Treatment including the possibility of a rescue i.v. dose was repeated upon each relapse.

The study was approved by the institutional review board/independent ethics committee and was performed in accordance with the Declaration of Helsinki. Written informed consent was obtained for all participants from parents or legal guardians and from patients, if appropriate.

### Assessments

At baseline and at each study visit (post-treatment Day 1, Day 2, Week 1 and Week 5 of each treatment period), physicians assessed global disease activity and rash using a 5-point scale: absent, minimal, mild, moderate or severe. Blood samples were collected for assessment of CRP and SAA (at each study visit) and to assess hematological and biochemical markers (baseline, post-treatment Day 1, Day 2, Week 1, Week 5 and thereafter monthly each period) and immunogenicity (baseline, 1 day pre-dose, and Week 5 of each period).

### Efficacy assessments

The primary efficacy variable was time-to-relapse after achieving a complete response. A complete response was defined as a global assessment of no or minimal disease activity and no or minimal rash, and CRP and/or SAA levels within the normal range (< 10 mg/L for both parameters). Relapse was defined as having a global assessment of disease activity of mild or greater or a global assessment of disease activity of minimal and an assessment of rash of mild or greater, plus CRP and/or SAA levels of > 30 mg/L. Alternatively, for patients with low CRP/SAA levels at baseline relapse was defined by a clinical picture necessitating retreatment, based on physician's global assessment of disease activity and rash. Secondary efficacy variables included the proportion of patients showing a complete response, physician assessments of disease activity, changes in levels of CRP and SAA.

### Pharmacokinetic assessments

Canakinumab concentrations were assessed in serum by competitive ELISA assay (lower limit of quantification (LLOQ) = 100 ng/mL). Pharmacokinetic parameters were determined by non-compartmental analysis. IL-1β levels were analyzed as previously described [13].

### Safety assessments

Adverse events (AEs) were monitored throughout the study. At each study visit, physicians asked patients for, or assessed (dosing visit), any local injection site reactions following s.c. administration. Other safety assessments included the regular monitoring of vital signs, hematology blood chemistry and urinalysis, and assays for anti-canakinumab antibodies using a binding BIAcore^® ^assay (Biacore International AB, Rapsgatan 7, 754 50 Uppsala, Sweden) [16]. Height and weight were measured at baseline and at the end of the study.

### Statistical analysis

A Weibull gap-time frailty model was used to estimate time-to-relapse for each dose regimen [17]. For patients who required an additional rescue dose of canakinumab, the dose regimen was defined as a separate group and the time-to-relapse was calculated from the time of i.v. rescue dose. Analyses were conducted using SAS software, version 8.2 (SAS Institute, Cary, NC, USA).

## Results

**Figure 1:**
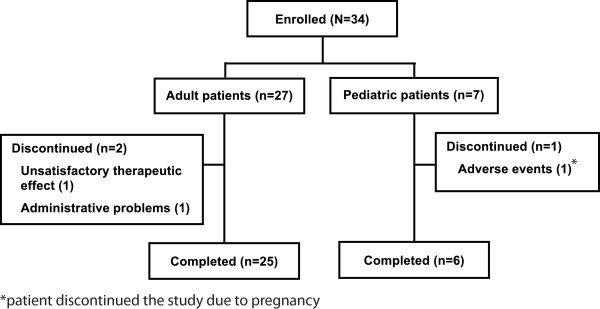
**Patient composition**.

A total of 34 patients were enrolled in this phase II, open-label study and the overall patient disposition is presented in (Figure 1). Here we report data for seven pediatric patients (five children aged 4 to 13 years with MWS and two adolescents, aged 16 and 17 years, with NOMID).

Demographics and baseline disease characteristics are summarized in table 1. Six patients had previously received anakinra and four had achieved a complete response; one other patient achieved a partial response and the sixth patient did not respond to anakinra. All five children received canakinumab at a dose of 2 mg/kg s.c. (with or without a rescue dose) while the two adolescents received canakinumab 150 mg s.c. All patients received at least one dose of canakinumab and the median number of doses received was six (range 1 to 20, including rescue doses). The total duration of exposure to canakinumab ranged from 126 to 463 days.

**Table 1:** Demographics and baseline disease characteristics

Patient	Clinical picture/NALP3mutation - Clinical symptom	Weight (kg)	CRP (mg/L)	SAA (mg/L)	Previous anakinra use/response	Physician's global assessment of disease activity
1	MWS/V198M - pyrexia, conjunctivitis, headache, abdominal pain, arthralgia and lassitude	17.2	8.2	1.8	Yes/No*	Moderate
2	MWS/E311K- aphthae, stomach pain, fatigue, loss of energy, headache, conjunctivitis, myalgia, fever peaks, beginning of hearing deficit and arthralgia during night.	18.1	2.0	2.1	Yes/Partial**	Moderate
3	MWS/T348M- Vomiting	23.3	39.0^#^	74.0^#^	Yes/Yes	Moderate
4	MWS/V198M - Low level of immunoglobulins, pyrexia, sensitivity to infection, coldness exposed exanthema, conjunctivitis, headache, oral aphthae, abdominal pain, myalgia and fatigue.	24.1	0.2	2.9	Yes/Yes	Moderate
5	MWS/E311K - exanthema, myalgia, conjunctivitis, attention deficit, headache, lack of concentration, oral aphthae, fatigue and hearing deficiency	35.3	9.9	14.1	No	Moderate
6†	NOMID/T348M - urticarial rash, hepatomegaly, pyrexia, anemia, headache, stomach pain, malaise, nausea, fatigue, conjunctivitis, high frequency hearing loss (1 KHz), sterile meningitis, papilloedema, growth retardation, bilateral reduced visual acuity, knee arthritis, back pain, myalgia, leucocytosis, and thrombocytosis	48.6	38.9	198.0	Yes/Yes	Moderate
7†	NOMID/G569R - exanthema, papillar edema, pseudo-tumor cerebri, hearing loss, arthritis, enlarged inner and outer liquor cavities, morphologically elevated bilateral intraocular pressure, and an enlarged blind spot in the visual field	52.0	65.6	151.0	Yes/Yes	Moderate

CRP, C-reactive protein; SAA, Serum Amyloid A

^# ^Local lab values

†The two adolescent patients had MWS/NOMID

*No response - lack of efficacy while on anakinra

**Partial response - MWS activity despite of anakinra therapy and necessity of anakinra dosage increase

### Efficacy

**Figure 2:**
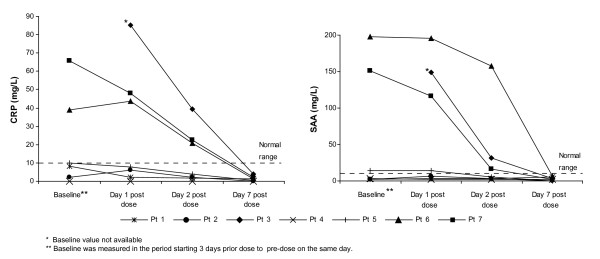
**Reduction in inflammatory markers in response to a single dose of canakinumab**.

All patients achieved a complete response to their first dose of canakinumab and this was achieved within one day in four patients and within seven days in the remaining three patients. Improvements in symptoms were evident in all patients within 24 hours of administration of canakinumab; at baseline all patients had moderate disease activity, while one day post-dose, disease activity was absent in four patients and minimal in the other three. CRP and SAA levels normalized within seven days in patients who had baseline values above the normal range (< 10 mg/L). The levels of CRP and SAA were maintained at normal levels in those patients who already had normal levels at baseline (Figure 2).

**Figure 3:**
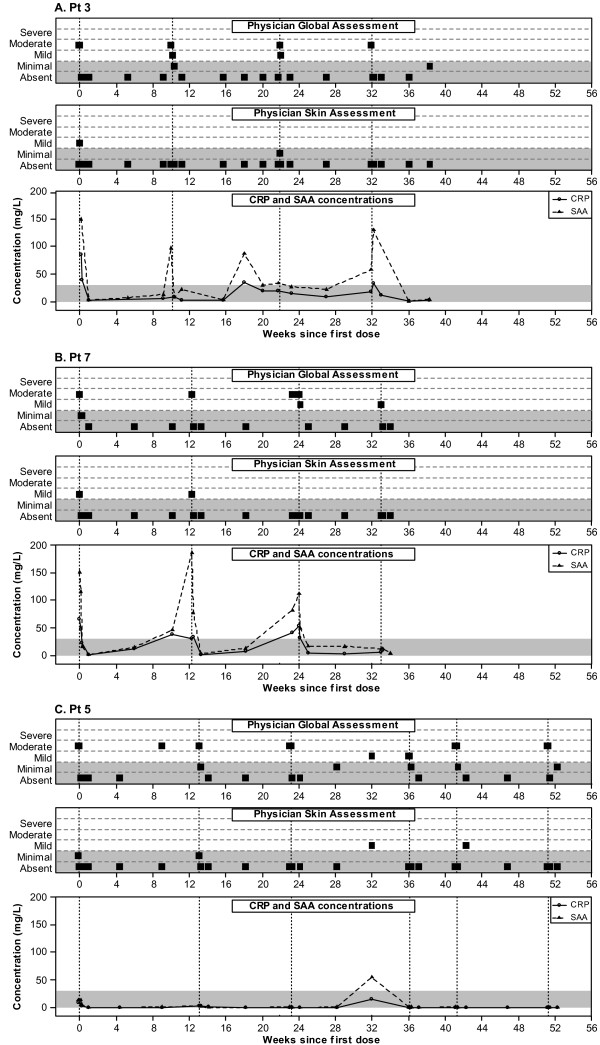
**Response pattern in three patients: physician global assessment, physician skin assessment and CRP and SAA levels**. In the upper and middle panels, the squares represent the physician's assessment of global disease activity and rash. The shaded areas indicate absent and mild severity. In the lower panel, the concentrations of CRP (solid line, circle) and SAA (dotted line, triangle) are presented. The shaded area indicates a concentration of 0 to 30 mg/L. Vertical dotted lines on all panels indicate the time of re-dosing.

Six patients were retreated on relapse and four achieved a second complete response within seven days post-treatment and continued to achieve complete responses on retreatment with single doses of canakinumab on most occasions. Clinical relapses (that is, increase in both global assessment and skin assessment to mild or greater) were typically accompanied by an increase in SAA and possibly CRP to greater than 30 mg/L and this pattern was observed in both children and adolescents (Figure 3A, B). In one patient, clinical relapses were not accompanied by increases in SAA and/or CRP on most occasions (Figure 3C).

All five children on canakinumab 2 mg/kg s.c. achieved a complete response within seven days post initial dose. However, two patients with the V198M mutation relapsed within seven days in many treatment cycles and needed an i.v. rescue to achieve and maintain complete response (a rescue dose was administered in one patient after each s.c. dose and for the other patient on three occasions out of nine treatments). In these two patients with V198M mutation, rash and conjunctivitis were absent; symptoms of headache, abdominal pain, myalgia, fatigue, and fever episodes in one patient were present, all being reversible.

The observed times to relapse for two adolescent patients receiving 150 mg s.c. were > 125 days in one who was discontinued after the first dose without experiencing a relapse and 86 days and 77 days in the second adolescent. Those values are in agreement with the median time to relapse of 115 days (95% CI 94.1 to 136.4) estimated using a Weibull analysis in the group of adults and adolescent patients receiving 150 mg s.c. For children receiving 2 mg/kg s.c. the estimated median time-to-relapse was 49 (95% CI: 29.3 to 67.9) days (Table 2).

**Table 2:** Estimated median time to relapse

Canakinumab Dose regimen	No. of subjects	No. of periods	Median time to relapse (days)	95% confidence interval
2 mg/kg s.c.	4	22	48.6	29.3 to 67.9
2 mg/kg s.c. + rescue i.v.	2**	11	51.7	27.0 to 76.5

** Two subjects received rescue medication for some periods. In one out of these two pediatric patients, data of periods in which rescue medication was not given were analyzed with the 2 mg/kg s.c. group, while data of periods in which rescue medication was given were analyzed separately with the 2 mg/kg s.c. + rescue i.v. group.

At study start, "fatigue", which was part of the core variables for the physicians' global assessment, was severe in two patients, moderate in three patients and mild in one patient. One day post-dose "fatigue" was absent in five patients and minimal in two patients and this was maintained until the patient experienced the next relapse.

### PK/PD and IL-1β levels

The pharmacokinetic parameters for canakinumab are summarized in Table 3. Peak concentrations of 7.7 to 13.6 μg/mL were achieved in children receiving doses of 2 mg/kg (total dose, 35 to 96 mg) after 2 to 7 days and the apparent half life of canakinumab was 23 to 26 days. The rates of IL-1β production for the five MWS patients were 20.1 (Pt 1), 6.02 (Pt 2), 21.2 (Pt 3), 4.5 (Pt 4), and 5.8 (Pt 5) ng/day, and for the two NOMID patients 9.6 (Pt 6) and 16.7 (Pt 7) ng/day.

**Table 3:** Pharmacokinetic parameters following administration of the first dose of 150 mg or 2 mg/kg canakinumab

Patient	Dose (mg)	*C*_max _(μg/mL)	*T*_max _(d)	AUC_0-∞ _(μgd/mL)	*t*_1/2 _(d)	*CL/F *(L/d)	*Vz/F *(L)	*CL/F/wt *(L/d)/kg
1	34.8	NE	NE	NE	NE	NE	NE	NE
2	36.0	13.6	6.96	580	25.7	0.0621	2.3	0.0034
3	46.6	7.67	2	NE	NE	NE	NE	NE
4	48.0	NE	NE	NE	NE	NE	NE	NE
5	71.0	12.4	2	543	23.7	0.131	4.48	0.0037
6	150.0	16.3	7.05	647	22.9	0.232	7.67	0.0048
7	150.0	10.4	2.16	NE	NE	NE	NE	NE

NE: : non-estimable

*C*_max_, Maximum (peak) serum drug concentration after drug administration; *T*_max_, Time to reach peak or maximum concentration following drug administration; AUC0∞, Area under the concentration-time curve from time zero to infinity; *t*_1/2_, The elimination half-life associated with the terminal slope (λz) of a semi logarithmic concentration-time curve; CL/F, The apparent total clearance from serum; Vz/F, The apparent volume of distribution during terminal phase

### Safety

Canakinumab was generally well tolerated. All AEs were of mild to moderate severity. One serious AE (SAE) was reported, a case of vertigo (V198M, MWS) that resolved during treatment. The most frequently reported AEs were upper respiratory tract infections and rash (Table 4). One patient, aged 16 years, discontinued due to positive pregnancy test (pregnancy test was negative at enrolment and baseline). The pregnant adolescent was still in remission until day 126 (time of discontinuing the study). After discontinuation, the patient did not receive anakinra, and prednisone treatment (40 mg at first, then 20 mg daily based on the investigator's assessment of disease condition) was started. Prednisone was partially effective without reaching serological remission (CRP levels 10 to 30 mg/L, SAA levels 13 to 75 mg/L). The pregnancy was uneventful and the newborn had a normal Apgar score at birth as well as all clinical findings were unremarkable. Injections were well tolerated. Three patients reported mild to moderate injection-site reactions; in total in six occasions from 54 injections. No anti-canakinumab antibodies were detected in any patients.

**Table 4:** Adverse events

Patients with AEs and SAEs	n (%)
SAEs*	1 (14)
**AEs leading to study drug discontinuation**	1**
**AEs reported in ≥2 patients**	
Upper respiratory tract infection	5 (71)
Rash	4 (57)
Pharyngitis	3 (43)
Nasopharyngitis	3 (43)
Vomiting	3 (43)
Diarrhea	2 (29)
Rhinitis	2 (29)
Sleep disorder	2 (29)
Cough	2 (29)
Pharyngolaryngeal pain	2 (29)
Acne	2 (29)

*One patient experienced vertigo, considered as a SAE, which resolved during treatment. ** pregnancy

Markers of systemic inflammation such as white blood cell count, neutrophil count and platelet count were at the upper limit of normal at baseline and decreased within the first 24 hours post-dose.

Two children were anemic at baseline, and all children had normal hemoglobin levels at the end of the study. All other laboratory or biochemical markers (glucose, albumin, creatinine, total protein, creatine kinase, triglycerides, total cholesterol, serum glutamic oxaloacetic transaminases, serum glutamic pyruvic transaminases, and gamma-glutamyl transferase) showed little change over the course of the study. No clinically relevant changes in diastolic and systolic blood pressure or pulse were observed.

Over the course of the study, children gained in height and weight. The four younger children (4 to 7 years) who were treated on the study for 9 to 15 months gained 2.1 to 5.6 kg and the two for whom height data were available gained 4 cm in height. The fifth child, aged 13, was treated in the study for 12 months and gained 12.6 kg in weight (from 35.3 kg at study entry) and 9 cm in height (from 155 cm at baseline).

## Discussion

The results reported here indicate that canakinumab is a highly effective and well tolerated therapy for the treatment of CAPS in pediatric patients. All patients achieved complete clinical and biochemical responses within seven days of receiving the first dose of canakinumab and responses were rapidly re-induced on re-treatment after relapse in most patients. In addition, canakinumab was well tolerated in all patients.

This study included children who were younger than those enrolled in the previously conducted study in CAPS patients (31 adults and 4 children) receiving canakinumab 150 mg s.c. (body weight ≥40 kg) or 2 mg/kg s.c. (body weight < 40 kg), administered every eight weeks [15] and the results observed in both these studies were comparable thus extending the observations to children as young as four years old. In both studies, responses to canakinumab were rapid with improvements in symptoms being observed within 24 hours of administration of the first dose in most patients. In addition, most patients achieved a complete response within one week following administration of a single dose. Responses were sustained in most patients and were re-induced with a single dose of canakinumab on re-treatment.

In this study, the pharmacokinetics of canakinumab in pediatric patients was found to be similar to that in adults [18]. The half-life of canakinumab in children ranged from 23 to 26 days, comparable to that previously reported for adults with rheumatoid arthritis (that is, 21 to 28 days) [14] and for adults with CAPS (26 days) [13,15]. This supports every eight-week dosing in children as in adults, which was shown to produce sustained remissions in the phase III study which included four children. This compares favourably with the very short half-life of anakinra (four to six hours) [19] and half-life of 6.3 days in children and 7 days in the adult population for rilonacept [20]. As a result of its very short half-life, anakinra is administered once daily while rilonacept is administered once weekly. This need for frequent injections is an important shortcoming of these medications, especially for children who may well be afraid of needles and do not understand the importance of their medication. The prolonged half-life of canakinumab allows sustained remissions to be achieved with eight-weekly dosing and, therefore, represents a major step forward in the management of this debilitating disorder.

The estimated median time-to-relapse in this study ranged from approximately 50 days in children receiving a dose of 2 mg/kg s.c. to 115 days in adolescents and adults receiving a dose of 150 mg s.c. Thus the estimated time-to-relapse in children was approximately half that for adults. This does not fit with the pharmacokinetic data for canakinumab reported for this study, which show the pharmacokinetics of canakinumab in pediatric patients to be similar to those in adults and suggests that factors other than pharmacokinetics of canakinumab may have contributed to the difference in time-to-relapse. Two children with CAPS symptoms did not have elevated CRP/SAA levels at baseline and were diagnosed for V198M. One of these patients failed to achieve complete response with a single s.c. dose on a number of occasions and both patients were frequently re-dosed (residual disease symptoms with anakinra treatment (dose increased over the first months in one patient and over the first year in second patient) and frequent necessity of dosage increase to anakinra was recorded in the medical history). V198M mutation has been described as inducing CAPS with a heterogeneous phenotype which variably responds to increasing doses of anti IL-1 medication [21-23]. In addition, Aksentijevich *et al*. reported a patient with a V198M variant who did not adequately respond to anakinra treatment and discussed that other so far unknown genetic factors may be involved in the disease phenotype [24]. Thus, it is possible that patients with particular mutations such as the V198M mutation may require higher doses of canakinumab or more frequent administration to maintain their response and have contributed to the lower value for time-to-relapse for children reported for this study.

In this study, six patients received anakinra treatment prior to enrolment. Four were complete responders, one patient responded partially and one patient failed to respond to anakinra therapy. The fact that both the partial and non-responder patients achieved a complete response to canakinumab is thus encouraging.

In addition to achieving sustained clinical remissions, most pediatric patients achieved sustained normalization of CRP and SAA levels. This is likely to have important implications for the long-term outcome of patients since prolonged elevation of SAA levels is associated with the development of AA amyloidosis which severely impairs renal function and leads to renal failure and death if effective therapy is not given. A recent study of patients with a variety of inflammatory conditions reported that the risk of death is increased 18-fold in patients with high SAA levels (> 155 mg/dL) compared with those with levels within the normal range [25]. In addition, a reduction in SAA levels and a regression of amyloid deposits was found to be associated with improved outcomes, suggesting that normalization of SAA levels in patients with CAPS is likely to significantly reduce their risk of renal failure.

All children showed rapid improvement of fatigue following treatment, which is a great advantage for their quality of life. Reduced fatigue leads to improved receptiveness in school and is a major advantage in development.

The data reported here indicate that canakinumab is well tolerated in children and adolescents, as well as in adults, as reported previously [15] and elsewhere for this study [13]. All adverse events were mild or moderate in severity and only one SAE, a case of vertigo that resolved during treatment, was reported. These data support those previously reported for the placebo-controlled phase of the phase III study [15]. In addition, in both studies [13,15] few patients reported injection site reactions and none of these were severe. This contrasts with anakinra and rilonacept; in studies in patients with CAPS, approximately half of patients experienced injection-site reactions and an increased risk of infections was observed [10,11,26]. In this study, children were found to gain in height and weight, suggesting that canakinumab by inhibiting the catabolic state linked to chronic inflammation allows normal development in the children.

The results of this study provide an important confirmation of the efficacy of canakinumab in children with this rare disorder and suggest that the safety profile of canakinumab in pediatric patients is similar to that in adults. Safety data for canakinumab in children have also been reported for doses of 0.5 to 9 mg/kg in children with systemic juvenile idiopathic arthritis (SJIA) and confirm the favourable safety profile of canakinumab in children [27].

However, the results reported here are necessarily limited since they are based on only seven pediatric patients in a non controlled study. Also, the definition of relapse remains elusive in auto-inflammatory disease purely on clinical grounds. Although the efficacy and safety of canakinumab for the treatment of pediatric CAPS patients is confirmed in this study, the long-term impact of canakinumab treatment on the course of the disease still needs to be addressed further. In addition, further research is warranted in CAPS patients who have the V198M mutation to understand why they require higher doses of canakinumab or more frequent administration to maintain their response.

Recently, canakinumab was approved by EMA for treatment of CAPS and by the FDA for FCAS and MWS in adult and pediatric patients [28,29], giving physicians an important new therapy for the management of this debilitating disorder. Early diagnosis and prompt initiation of treatment should enable children with this rare disorder to live a more normal life and may reduce the risk of long-term sequelae, such as progressive loss of vision and hearing and development of renal insufficiency.

## Conclusions

Canakinumab is an effective, well tolerated therapy for pediatric patients with CAPS.

## Abbreviations

AEs: adverse events; CAPS: cryopyrin-associated periodic syndrome; CINCA: chronic infantile neurological cutaneous and articular syndrome; CRP: serum C-reactive protein; FCAS: familial cold auto-inflammatory syndrome; i.v.: intravenously; IL-1β: interleukin-1β; LLOQ: lower limit of quantification; MWS: Muckle-Wells syndrome; NOMID: neonatal-onset multisystem inflammatory disease; s.c.: subcutaneously; SAA: serum amyloid A protein; SAEs: serious adverse events; SJIA: systemic juvenile idiopathic arthritis.

## Competing interests

JKD is a consultant for Novartis Pharma and has received research grants and honorarium for lectures/symposiums. NB is a consultant for Novartis Pharma. SDF, TJ, KS, AC, ST, AMW and CR are employees of Novartis Pharma and own stock options in the company, and the company holds patents for the drug molecule.

## Authors' contributions

JBK-D, CR, SDF, and AC contributed to study conception and design, acquisition of data, analysis and interpretation of data, drafting the article or revising it critically for important intellectual content, and approval of the version of the article submitted for publication. CR was the translational medicine expert and SDF the clinical trial leader for this study. AMW was the trial statistician and contributed to study conception and design, analysis and interpretation of data, revising the article critically for important intellectual content, and approval of the version of the article submitted for publication. ST contributed to analysis and interpretation of data, revising the article critically for important intellectual content, and approval of the version of the article submitted for publication. JR contributed to acquisition of data, analysis and interpretation of data, revising the article critically for important intellectual content, and approval of the version of the article submitted for publication. NB and ER contributed to acquisition of data, revising the article critically for important intellectual content, and approval of the version of the article submitted for publication. TJ contributed to the study conception and design, analysis and interpretation of data, and approval of the version of the article submitted for publication. KS contributed to the interpretation of data, revising the article critically for important intellectual content, and approval of the version of the article submitted for publication.

## References

[B1] Hoffman HM Mueller JL Broide DH Wanderer AA Kolodner RD Mutation of a new gene encoding a putative pyrin-like protein causes familial cold autoinflammatory syndrome and Muckle-Wells syndrome Nat Genet 2001 29 301 305 10.1038/ng756 11687797 PMC4322000

[B2] Cinca syndrome http://www.orpha.net/data/patho/GB/uk-cinca.pdf

[B3] Farasat S Aksentijevich I Toro JR Autoinflammatory diseases: clinical and genetic advances Arch Dermatol 2008 144 392 402 10.1001/archderm.144.3.392 18347298

[B4] Glaser RL Goldbach-Mansky R The spectrum of monogenic autoinflammatory syndromes: understanding disease mechanisms and use of targeted therapies Curr Allergy Asthma Rep 2008 8 288 298 10.1007/s11882-008-0047-1 18606080 PMC2735099

[B5] Touitou I Koné-Paut I Autoinflammtory diseases Best Pract Res Clin Rheumatol 2008 22 811 829 10.1016/j.berh.2008.08.009 19028365

[B6] Aganna E Martinon F Hawkins PN Ross JB Swan DC Booth DR Lachmann HJ Bybee A Gaudet R Woo P Feighery C Cotter FE Thome M Hitman GA Tschopp J McDermott MF Association of mutations in the NALP3/CIAS1/PYPAF1 gene with a broad phenotype including recurrent fever, cold sensitivity, sensorineural deafness, and AA amyloidosis Arthritis Rheum 2002 46 2445 2452 10.1002/art.10509 12355493

[B7] Dode C Le Du N Cuisset L Letourneur F Berthelot JM Vaudour G Meyrier A Watts RA Scott DG Nicholls A Granel B Frances C Garcier F Edery P Boulinguez S Domergues JP Delpech M Grateau G New mutations of CIAS1 that are responsible for Muckle-Wells syndrome and familial cold urticaria: a novel mutation underlies both syndromes Am J Hum Genet 2002 70 1498 1506 10.1086/340786 11992256 PMC379138

[B8] Dinarello CA The many worlds of reducing interleukin-1 Arthritis Rheum 2005 52 1960 1967 10.1002/art.21107 15986340

[B9] Sutterwala FS Ogura Y Szczepanik M Lara-Tejero M Lichtenberger GS Grant EP Bertin J Coyle AJ Galan JE Askenase PW Flavell RA Critical role for NALP3/CIAS1/Cryopyrin in innate and adaptive immunity through its regulation of caspase-1 Immunity 2006 24 317 327 10.1016/j.immuni.2006.02.004 16546100

[B10] Goldbach-Mansky R Dailey NJ Canna SW Gelabert A Jones J Rubin BI Kim HJ Brewer C Zalewski C Wiggs E Hill S Turner ML Karp BI Aksentijevich I Pucino F Penzak SR Haverkamp MH Stein L Adams BS Moore TL Fuhlbrigge RC Shaham B Jarvis JN O'Neil K Vehe RK Beitz LO Gardner G Hannan WP Warren RW Horn W Neonatal-onset multisystem inflammatory disease responsive to interleukin-1beta inhibition N Engl J Med 2006 355 581 592 10.1056/NEJMoa055137 16899778 PMC4178954

[B11] Hoffman HM Throne ML Amar NJ Sebai M Kivitz AJ Kavanaugh A Weinstein SP Belomestnov P Yancopoulos GD Stahl N Mellis SJ Efficacy and safety of rilonacept (interleukin-1 Trap) in patients with cryopyrin-associated periodic syndromes: results from two sequential placebo-controlled studies Arthritis Rheum 2008 58 2443 2452 10.1002/art.23687 18668535

[B12] Ramos E Arostegui JI Campuzano S Rius J Bousono C Yague J Positive clinical and biochemical responses to anakinra in a 3-yr-old patient with cryopyrin-associated periodic syndrome (CAPS) Rheumatology (Oxford) 2005 44 1072 1073 10.1093/rheumatology/keh652 15840596

[B13] Lachmann HJ Lowe P Felix SD Rordorf C Leslie K Madhoo S Wittkowski H Bek S Hartmann N Bosset S Hawkins PN Jung T *In vivo *regulation of interleukin 1beta in patients with cryopyrin-associated periodic syndromes J Exp Med 2009 206 1029 1036 10.1084/jem.20082481 19364880 PMC2715040

[B14] Alten R Gram H Joosten LA van den Berg WB Sieper J Wassenberg S Burmester G van Riel P Diaz-Lorente M Bruin GJ Woodworth TG Rordorf C Batard Y Wright AM Jung T The human anti-IL-1 beta monoclonal antibody ACZ885 is effective in joint inflammation models in mice and in a proof-of-concept study in patients with rheumatoid arthritis Arthritis Res Ther 2008 10 R67 10.1186/ar2438 18534016 PMC2483458

[B15] Lachmann HJ Kone-Paut I Kuemmerle-Deschner JB Leslie KS Hachulla E Quartier P Gitton X Widmer A Patel N Hawkins PN Use of canakinumab in the cryopyrin-associated periodic syndrome N Engl J Med 2009 360 2416 2425 10.1056/NEJMoa0810787 19494217

[B16] Mire-Sluis AR Barrett YC Devanarayan V Koren E Liu H Maia M Parish T Scott G Shankar G Shores E Swanson SJ Taniguchi G Wierda D Zuckerman LA Recommendations for the design and optimization of immunoassays used in the detection of host antibodies against biotechnology products J Immunol Methods 2004 289 1 16 10.1016/j.jim.2004.06.002 15251407

[B17] Duchateau L Janssen PP The Frailty Model 2008 New York: Springer

[B18] Lachmann HJ Tannenbaum S Chakraborty A Preiss R Hawkins PN Pharmacokinetics (PK) of canakinumab (ACZ885, a fully human anti-IL-1beta monoclonal antibody) in cryopyrin-associated periodic fever syndrome (CAPS) patients [abstract] Ann Rheum Dis 2009 68 196 10.1136/ard.2007.086009 18385276

[B19] Burger D Dayer JM Palmer G Gabay C Is IL-1 a good therapeutic target in the treatment of arthritis? Best Pract Res Clin Rheumatol 2006 20 879 896 10.1016/j.berh.2006.06.004 16980212

[B20] Gillespie J Mathews R McDermott MF Rilonacept in the management of cryopyrin-associated periodic syndromes (CAPS) J Inflamm Res 2010 3 1 8 22096352 10.2147/jir.s8109 PMC3218731

[B21] Porksen G Lohse P Rosen-Wolff A Heyden S Forster T Wendisch J Heubner G Bernuth H Sallmann S Gahr M Roesler J Periodic fever, mild arthralgias, and reversible moderate and severe organ inflammation associated with the V198M mutation in the CIAS1 gene in three German patients--expanding phenotype of CIAS1 related autoinflammatory syndrome Eur J Haematol 2004 73 123 127 10.1111/j.1600-0609.2004.00270.x 15245511

[B22] Touitou I Perez C Dumont B Federici L Jorgensen C Refractory auto-inflammatory syndrome associated with digenic transmission of low-penetrance tumour necrosis factor receptor-associated periodic syndrome and cryopyrin-associated periodic syndrome mutations Ann Rheum Dis 2006 65 1530 1531 10.1136/ard.2006.054312 17038455 PMC1798361

[B23] Rosen-Wolff A Quietzsch J Schroder H Lehmann R Gahr M Roesler J Two German CINCA (NOMID) patients with different clinical severity and response to anti-inflammatory treatment Eur J Haematol 2003 71 215 219 10.1034/j.1600-0609.2003.00109.x 12930324

[B24] Aksentijevich I Putnam DC Remmers EF Mueller JL Le J Kolodner RD Moak Z Chuang M Austin F Goldbach-Mansky R Hoffman HM Kastner DL The clinical continuum of cryopyrinopathies: novel CIAS1 mutations in North American patients and a new cryopyrin model Arthritis Rheum 2007 56 1273 1285 10.1002/art.22491 17393462 PMC4321998

[B25] Lachmann HJ Goodman HJ Gilbertson JA Gallimore JR Sabin CA Gillmore JD Hawkins PN Natural history and outcome in systemic AA amyloidosis N Engl J Med 2007 356 2361 2371 10.1056/NEJMoa070265 17554117

[B26] Ross JB Finlayson LA Klotz PJ Langley RG Gaudet R Thompson K Churchman SM McDermott MF Hawkins PN Use of anakinra (Kineret) in the treatment of familial cold autoinflammatory syndrome with a 16-month follow-up J Cutan Med Surg 2008 12 8 16 18258152 10.2310/7750.2008.07050

[B27] Ruperto N Quartier P Wulffraat P Woo P Loy A Mouy A Bader-Meunier B Prakken B Noseda E Belleli R Lecot J Rordorf C Martini AfP A phase II trial with canakinumab (ACZ885), a new IL-1-beta blocking monoclonal antibody, to evaluate safety and preliminary efficacy in children with systemic juvenile idiopathic arthritis (SJIA) [abstract] Ann Rheum Dis 2009 68 170

[B28] Committee for medicinal products for human use: Summary of positive opinion for Ilaris http://www.ema.europa.eu/docs/en_GB/document_library/Summary_of_opinion_-_Initial_authorisation/human/001109/WC500059888.pdf

[B29] cder Drug and Biologic Calendar Year Approvals http://www.fda.gov/downloads/Drugs/DevelopmentApprovalProcess/HowDrugsareDevelopedandApproved/DrugandBiologicApprovalReports/PriorityNDAandBLAApprovals/UCM090995.pdf

